# ER as master regulator of membrane trafficking and organelle function

**DOI:** 10.1083/jcb.202205135

**Published:** 2022-09-15

**Authors:** Eva Maria Wenzel, Liv Anker Elfmark, Harald Stenmark, Camilla Raiborg

**Affiliations:** 1 Centre for Cancer Cell Reprogramming, Faculty of Medicine, University of Oslo, Oslo, Norway; 2 Department of Molecular Cell Biology, Institute for Cancer Research, Oslo University Hospital, Oslo, Norway

## Abstract

The endoplasmic reticulum (ER), which occupies a large portion of the cytoplasm, is the cell’s main site for the biosynthesis of lipids and carbohydrate conjugates, and it is essential for folding, assembly, and biosynthetic transport of secreted proteins and integral membrane proteins. The discovery of abundant membrane contact sites (MCSs) between the ER and other membrane compartments has revealed that, in addition to its biosynthetic and secretory functions, the ER plays key roles in the regulation of organelle dynamics and functions. In this review, we will discuss how the ER regulates endosomes, lysosomes, autophagosomes, mitochondria, peroxisomes, and the Golgi apparatus via MCSs. Such regulation occurs via lipid and Ca^2+^ transfer and also via control of in trans dephosphorylation reactions and organelle motility, positioning, fusion, and fission. The diverse controls of other organelles via MCSs manifest the ER as master regulator of organelle biology.

## Teaser

Wenzel et al. review how the endoplasmic reticulum controls the positioning, dynamics, and functions of other organelles via membrane contact sites.

## Introduction

The endoplasmic reticulum (ER) is the cell’s largest organelle and Ca^2+^ reservoir with well-characterized roles in the biosynthesis of lipids, proteins, and glycoconjugates. The more recent discoveries of membrane contact sites (MCSs) between the ER and other organelles have revealed that the functions of the ER go far beyond biosynthesis. Here, we will review these “non-traditional” functions of the ER. Since the interplays between the ER and the plasma membrane and lipid droplets have been extensively reviewed (Crul and Maleth, 2021; Renne and Hariri, 2021), we will focus on the interactions between the ER and intracellular organelles with emphasis on molecular mechanisms that control membrane trafficking and organelle function.

## ER in control of endosomes

**Figure 1.**
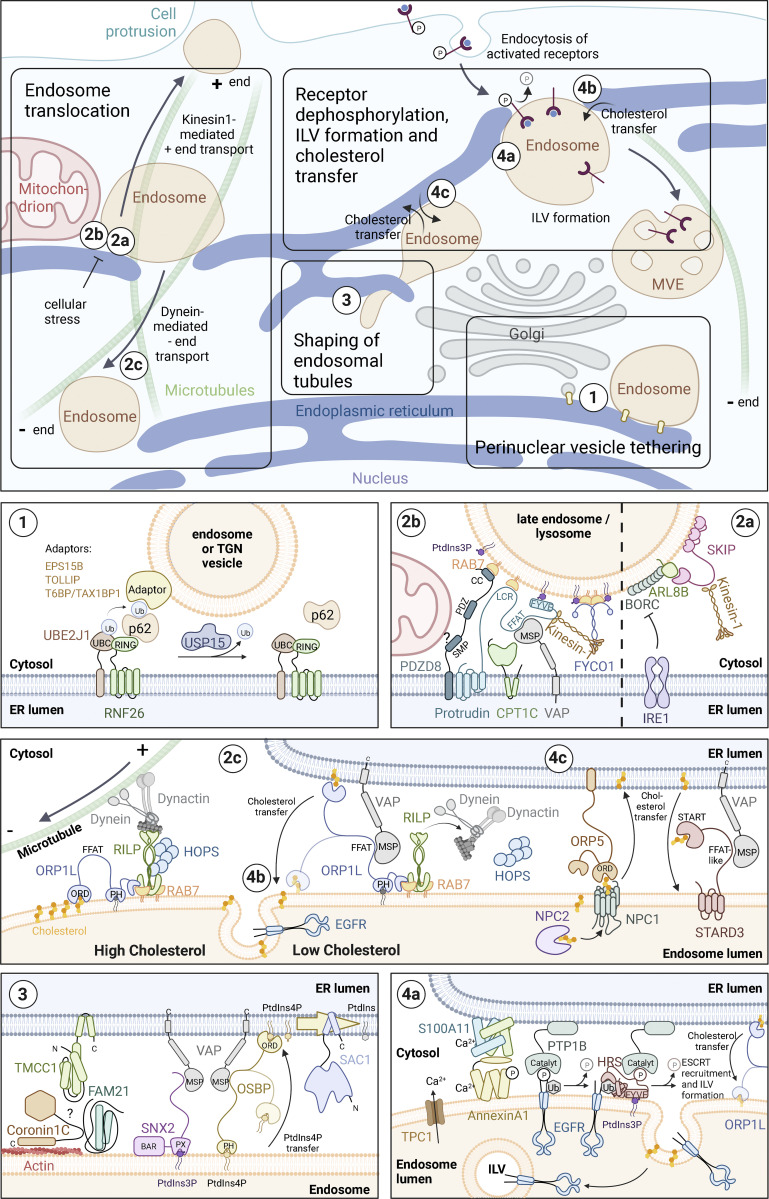
**ER-mediated control of endosome dynamics.** Overview of cell biological functions of ER-endosome contact sites and the involved molecules. The molecular composition of ER–endosome contact sites. OSBP, ORPs, and VAPs function as dimers or multimers. For simplicity, this is not displayed in the figure. VAP family members (see text box) are depicted as “VAP.” **(1)** Perinuclear vesicle tethering: The E2 ubiquitin-conjugating enzyme UBE2J1 activates the E3 ubiquitin ligase RNF26, which then ubiquitinates SQSTM1/p62. Ubiquitinated SQSTM1/p62 in turn binds to organelle-specific adaptor proteins, such as T6BP/TAX1BP1, on TGN vesicles and EPS15B or TOLLIP on endosomes. The release of the tethered vesicles is mediated by the deubiquitinase USP15. **(2)** Endosome translocation. **(2a)** The BORC complex recruits the small GTPase ARL8B to endosomes, which in turn recruits and activates the Kinesin-1 adaptor protein SKIP/PLEKHM2, resulting in plus-end directed movement of endosomes and lysosomes. Upon ER stress, IRE1 inhibits BORC-dependent anterograde endosome translocation. **(2b)** The ER-resident protein Protrudin contacts endosomes by binding to RAB7 and PtdIns3P. At these contact sites, Protrudin mediates the hand-over of Kinesin-1 to the endosomal adaptor protein FYCO1, allowing plus-end translocation of endosomes along microtubules. The activity of Protrudin can be regulated by CPT1C, which promotes anterograde endosome transport under nutrient-rich conditions and blocks it under cellular stress conditions. PDZD8 interacts with Protrudin and RAB7, also mediating ER-endosome contact. In addition, PDZD8 might mediate contact with mitochondria. **(2c)** Endosomes containing high levels of cholesterol move along microtubules in the minus-end direction by dynein/dynactin motor proteins, which connect to the endosome through RILP, RAB7, and ORP1L. Under low concentrations of cholesterol, ORP1L makes contact with VAP in the ER, which leads to the dissociation of dynein/dynactin and the HOPS complex. ER-endosome contact enables ORP1L to transfer cholesterol from the ER to endosomes. Sufficient levels of endosomal cholesterol are a prerequisite for ILV formation (see also legend to 4b). **(3)** Shaping of endosomal tubules: The formation of recycling tubules requires transient accumulation of PtdIns4P on endosomes to allow WASH-dependent actin nucleation and retromer function. OSBP interacts with PtdIns4P on endosomes via its PH domain and tethers endosomes to the ER via interaction with VAP. PtdIns4P is then dephosphorylated by the ER-resident lipid phosphatase SAC1, securing a transient PtdIns4P pool on endosomes. WASH is linked to the retromer by its subunit FAM21, which marks the site of tubule scission. The PtdIns3P-binding retromer subunit SNX2 is also able to interact with the ER through VAP. The ER protein TMCC1 and Coronin 1C on endosomes are required for contact site formation and fission of WASH-containing endosome tubules. It is not known whether Coronin 1C and TMCC1 interact directly, or if there are additional proteins required to generate these membrane contact sites. **(4)** Receptor dephosphorylation, ILV formation, and cholesterol transfer. **(4a)** EGFR-induced phosphorylation of Annexin A1 induces the formation of Annexin A1/S100A11-mediated ER-endosome contact sites, aided by the local increase in Ca^2+^ through the endosomal Ca^2+^ channel TPC1. PTP1B in the ER dephosphorylates EGFRs and ESCRT-0, facilitating the sorting of EGFRs into forming ILVs. **(4b)** In addition, Annexin A1/S100A11-mediated ER-endosome contact sites facilitate cholesterol transfer from ER to forming ILVs by ORP1L (see also legend to 2c). **(4c)** STARD3 and its paralog STARD3NL (not shown) mediate cholesterol transfer from ER to EGFR-negative endosomes. ORP5 facilitates cholesterol transport from endosomal membranes to the ER. The cholesterol is provided by NPC2 and NPC1, which interacts with ORP5, forming an ER–endosome contact. Direct shuttling of sterols using the ORD domain of ORP5 remains to be confirmed (Santos et al., 2020).

The endocytic pathway consists of numerous endocytic vesicles, endosomes, and lysosomes that receive the material taken up from the cell surface via endocytosis, including cargos such as nutrient receptors and activated growth factor and hormone receptors. Endocytic vesicles derived from the plasma membrane fuse with early endosomes, which mature and change their molecular composition as they move toward the cell interior guided by dynein-dependent transport along microtubules. As endosomes mature, they become gradually more acidic and acquire hydrolytic enzymes supplied by fusion with Golgi-derived vesicles. Finally, the resulting late endosomes fuse with lysosomes and their cargo is degraded (Huotari and Helenius, 2011; Scott et al., 2014). Although the endocytic and biosynthetic pathways have traditionally been considered to be highly separate, recent studies have revealed surprising connections between the ER and endosomes (Fig. 1).

### Perinuclear retention of endosomes

The bulk of endosomes and lysosomes exhibit a perinuclear localization clustered around the microtubule-organizing center, together with vesicles of the trans-Golgi network (TGN). This localization enables efficient endosome maturation and cargo trafficking, important for endocytic pathway functions including nutrient uptake, receptor downregulation and cell signaling, host defense against pathogens, and control of cell polarity and cell migration (Alanko et al., 2016; Huotari and Helenius, 2011; Scott et al., 2014). Although the perinuclear clustering of endosomes and Golgi vesicles has been observed for decades, how they are organized and retained was not understood until recently. The ER plays a direct role in the maintenance of this endosomal architecture, orchestrated by two ER-resident ubiquitination enzymes (Cremer et al., 2021; Jongsma et al., 2016; Fig. 1, 1). The E2 ubiquitin conjugation enzyme UBE2J1 interacts with and activates the multimembrane spanning RING domain E3 ubiquitin ligase RNF26. This induces the recruitment and ubiquitination of SQSTM1/p62, a cytosolic ubiquitin adapter best known for its role in selective autophagy. Ubiquitinated SQSTM1/p62 in turn interacts with many ubiquitin-binding organelle-specific adaptor proteins, including T6BP/TAX1BP1 at the TGN (Morriswood et al., 2007) and EPS15B or TOLLIP on endosomes (Katoh et al., 2004; Roxrud et al., 2008). The localization of the E2/E3 pair UBE2J1/RNF26 is confined to the perinuclear ER, which ensures the perinuclear retention of vesicles until released. The ubiquitin-dependent vesicle tethering is released by the deubiquitination enzyme USP15, which is recruited by RNF26 (Jongsma et al., 2016). Although the perinuclear retention of RNF26 depends on its RING domain, it is not known how this mechanism is regulated or how it is coordinated with mechanisms that translocate vesicles to the cell periphery. Dysregulation of ER-UBE2J1/RNF26-mediated vesicle tethering leads to the increased half-life of phosphorylated epidermal growth factor receptors (EGFR) accompanied by prolonged AKT-S473 phosphorylation due to impaired endocytic downregulation (Cremer et al., 2021).

### Regulation of endosome translocation to the cell periphery

The nutritional status as well as cellular stress responses influence how endosomes are positioned and utilized for cellular functions (Korolchuk et al., 2011; Raiborg, 2018). During stress and low nutrient conditions, endosomes and lysosomes cluster perinuclearly to facilitate cargo degradation for nutrient supply. When nutrients are available and in the absence of cellular stress, motile endosomes engage in a variety of cellular processes to support cell growth and development. The motile and dispersed late endosomes are less acidic and contain lesser hydrolytic enzymes than the perinuclear late endosomes (Johnson et al., 2016), consistent with their role in functions other than cargo degradation. Indeed, although some endosomes recycle cargo back to the cell surface, others are engaged in plasma membrane repair, protrusion formation, mTORC1 signaling, or secretion of exosomes (Ballabio and Bonifacino, 2020; Pu et al., 2016). Importantly many of these responses are coordinated through the ER. There are two established mechanisms that facilitate the centrifugal transport of late endosomes: the protrudin-dependent pathway, whose function depends on ER-resident proteins, and the BORC-dependent pathway, which is inhibited by ER stress (see below). Thus, the ER is a master regulator of endosome positioning through the control of mechanisms that promote their perinuclear or peripheral localization.

### Inhibition of BORC-dependent endosome translocation upon cellular stress

The eight-subunit protein BLOC-one-related complex (BORC) localizes to late endosomes. When nutrient supplies are rich, BORC recruits the small GTPase ARL8B, which through its effector SKIP/PLEKHM2 engages the plus-end-directed microtubule motor Kinesin-1, thus promoting late endosome translocation to the cell periphery (Fig. 1, 2 a). This mechanism is important for cell migration and axonal growth (Farías et al., 2017; Pu et al., 2015). Under cellular stress conditions, however, this pathway is turned off. When cells are deprived of amino acids and growth factors, the BORC complex binds to the endosomal Ragulator complex, making it unable to engage Kinesin-1 (Filipek et al., 2017; Pu et al., 2017). In addition, the ER-resident transmembrane nuclease inositol requiring enzyme 1 (IRE1) plays a role in shutting off BORC-dependent endosome translocation. One branch of the unfolded protein response triggered by ER stress goes through the activation of IRE1. Once activated, IRE1 cleaves and initiates the degradation of certain mRNAs, including the mRNA encoding Blos1, a subunit of the BORC complex. Thus, endosomes cluster perinuclearly, facilitating the lysosomal degradation and clearance of ubiquitinated protein aggregates by microautophagy during ER stress (Bae et al., 2019).

### Protrudin-mediated endosome translocation

Protrudin is a transmembrane ER-resident protein that induces ER–endosome MCSs by binding to the late endosomal small GTPase RAB7 in combination with the endosomally enriched lipid phosphatidylinositol 3-phosphate (PtdIns3P; see text box for RAB GTPases and phosphoinositides). In such MCSs, Protrudin hands over Kinesin-1 to the endosomal adapter protein FYCO1, which also interacts with RAB7 and PtdIns3P. This facilitates the translocation of late endosomes along microtubules to the plasma membrane (Raiborg et al., 2015a; Fig. 1, 2 b). The ER-resident pseudoenzyme carnitine palmitoyltransferase 1C (CPT1C) is found in a complex with Protrudin and functions as a nutrient sensor. Under glucose-rich conditions, malonyl-CoA binds CPT1C, and this activates Protrudin-mediated Kinesin-1 handover, which is inhibited upon cellular stress by signaling from the 5' AMP-activated protein kinase (AMPK; Palomo-Guerrero et al., 2019). It is not clear if the seemingly parallel BORC and Protrudin pathways are redundant. As they depend on different small GTPases, ARL8B and RAB7, respectively, they likely translocate different subpopulations of late endosomes (Jongsma et al., 2020). The Protrudin pathway is important for the formation of cellular protrusions like neurites or invadopodia, and this requires that endosomes fuse with the plasma membrane in a Synaptotagmin-VII-dependent manner (Palomo-Guerrero et al., 2019; Pedersen et al., 2020; Raiborg et al., 2015a; Shirane and Nakayama, 2006). In addition, the endosomes contain cargo, such as the metalloprotease MT1-MMP, and the overexpression of Protrudin increases the cell’s invasive behavior by facilitating exocytosis of MT1-MMP in growing invadopodia (Pedersen et al., 2020). Moreover, the Protrudin pathway facilitates mTORC1 signaling from late endosomes (Hong et al., 2017) and stimulates angiogenesis (Arora et al., 2022) and axon regeneration (Petrova et al., 2020).

The vesicle-associated membrane protein-associated protein (VAP) family consists of five dimeric transmembrane ER proteins that contain a major sperm protein (MSP) domain, which binds FFAT (two phenylalanines in an acidic tract) or FFNT (two phenylalanines in a neutral tract) motifs present in proteins on the membranes of various organelles to form MCSs (Cabukusta et al., 2020; James and Kehlenbach, 2021; Loewen and Levine, 2005). Mammalian VAPs include the FFAT-binding VAP-A, VAP-B, and MOSPD2, and the FFNT-binding MOSPD1 and MOSPD3.

RAB GTPases are small GTPases of the RAS superfamily, which act as molecular switches that are active in the GTP-bound form and inactive in the GDP-bound form (Stenmark, 2009). In their active conformation, RAB GTPases control membrane dynamics and intracellular transport by binding various effector proteins, including vesicle tethers, enzymes, and motor adaptors. Almost 70 different mammalian RAB GTPases have been identified, and they are known to associate with specific membranes such as the Golgi (RAB6), early endosomes (RAB5), or late endosomes/lysosomes (RAB7). Membrane association is mediated via C-terminal isoprenoid groups.

Phosphoinositides (PIs) are phosphorylated derivatives of the abundant membrane phospholipid, phosphatidylinositol (PtdIns; Schink et al., 2016). Seven PIs exist in nature – PtdIns3P, PtdIns4P, PtdIns5P, PtdIns(3,4)P_2_, PtdIns(3,5)P_2_, PtdIns(4,5)P_2_, and PtdIns(3,4,5)P_3_, with numbers indicating the positions of phosphates in the inositol headgroup. PtdIns3P, PtdIns4P, and PtdIns(4,5)P_2_ have been implicated in MCS formation and dynamics. Phosphorylations of the headgroup are mediated by isoform-specific PI kinases whereas dephosphorylations are catalyzed by specific PI phosphatases.

ORP (oxysterol binding protein-related protein) is a family of proteins that has the capacity to bind and transfer sterols and phosphoinositides (Nakatsu and Kawasaki, 2021). ORPs are characterized by an OSBP-related domain, ORD, which contains a hydrophobic sterol binding pocket. Most ORPs also contain phosphoinositide-binding pleckstrin homology (PH) domains and FFAT motifs, which mediate their localization and functions at MCSs.

### Synchronization of endosome translocation and lipid transfer

Lipid transfer between closely opposed organelles is mediated by lipid transfer proteins (Reinisch and Prinz, 2021). The ER-resident PDZ domain containing protein 8 (PDZD8) harbors lipid transfer activity and transfers glycerophospholipids and ceramide between membranes in vitro by the use of its synaptotagmin-like mitochondrial-lipid-binding (SMP) domain (Gao et al., 2022; Shirane et al., 2020). In vivo, the depletion of PDZD8 results in a decrease in the abundance of phosphatidylserine (PS) in neuronal endosomes (Shirane et al., 2020) and the accumulation of endosomal PtdIns(4,5)P_2_ (Jeyasimman et al., 2021). PDZD8 is important for endosome maturation and their degradative capacity, neuronal integrity, and neurite outgrowth (Gao et al., 2022; Jeyasimman et al., 2021; Shirane et al., 2020).

PDZD8 mediates ER–endosome contact sites by binding to RAB7 and interacts with Protrudin via its transmembrane domain (Elbaz-Alon et al., 2020; Gao et al., 2022; Guillén-Samander et al., 2019; Khan et al., 2021; Shirane et al., 2020; Fig. 1, 2 b). The potential functional relationship between Protrudin and PDZD8 is not completely understood. Since both proteins can form ER–endosome contact sites, why would they need to interact? It is tempting to speculate that these proteins cooperate in the regulation of endosome maturation and translocation, PDZD8, by mediating lipid transfer, and Protrudin by providing a microtubule motor protein. Thus, endosome maturation, function, and translocation can be coordinated efficiently by the ER. This might be especially important in neurons, which depend heavily on endosomal trafficking for their function.

### Coordination of microtubule-mediated retrograde and anterograde endosome transport

Endosome positioning entails a constant balance between minus- and plus-end-directed transport along microtubules, mediated by dynein or kinesins, respectively (Bonifacino and Neefjes, 2017; Gennerich and Vale, 2009). With its widespread connection to endosomes, the ER constitutes a unique platform for the organization of the required motor proteins (Friedman et al., 2013). One such possible coordination point centers on the ER-resident protein VAP-A (see text box). Despite being a transmembrane ER protein, Protrudin harbors a VAP-binding FFAT motif, and VAP-A is important for the proper distribution of Protrudin in the ER and for the function of Protrudin in protrusion formation, suggesting that VAP-A facilitates Kinesin-1-dependent endosome translocation (Saita et al., 2009; Fig. 1, 2 b). VAP-A is also implicated in the loss of dynein from endosomal membranes. The dynein binding endosomal protein RILP forms a tripartite complex with RAB7 and the endosomal cholesterol sensor ORP1L, a member of the ORP family (see text box). Under low endosomal cholesterol concentration, the endosomes become tethered to the ER by ORP1L binding to VAP-A, leading to the dissociation of dynein from RILP (Rocha et al., 2009; Fig. 1, 2 c). Thus, although not yet experimentally verified, it is conceivable that VAP-A sites in the ER coordinate the loss of endosomal dynein with the gain of Kinesin-1 through ORP1L-RILP and Protrudin.

Another clue to the role of ER as a coordinator of endosomal motor protein switching comes from the association between Protrudin and the long M1 isoform of the microtubule-severing AAA-ATPase, Spastin. Spastin interacts with Protrudin in the ER and inhibits Protrudin-dependent polarized membrane traffic (Connell et al., 2020). The inhibitory effect of Spastin on endosome translocation requires its ability to interact with the endosomal-sorting complex required for transport (ESCRT)-III proteins, IST1 and CHMP1B, in addition to its microtubule severing-activity. Although not completely understood, this effect might be related to the role of Spastin in the fission of endosomal recycling tubules, which requires the same functional properties as Spastin (Allison et al., 2013). The recruitment of dynein to Spastin-induced microtubule plus ends (Fassier et al., 2013; Lenz et al., 2006; Riano et al., 2009; Zhang et al., 2003) likely counteracts the Protrudin-mediated Kinesin-1-dependent movement of endosomes on microtubule rails toward the cell periphery (Wassmer et al., 2009). The interaction between Spastin and Protrudin in the ER could ensure that the microtubule severing is positioned in close proximity to Protrudin. Thus, the ER coordinates the recruitment of dynein and Kinesin-1 via Spastin and Protrudin, respectively.

### Shaping of endosomal tubules

Endocytic cargo that is not destined for lysosomal degradation is sorted into endosomal membrane tubules for recycling back to the plasma membrane or to the Golgi (Huotari and Helenius, 2011; Scott et al., 2014). This process involves membrane budding, tubule extension, and fission to generate cargo-containing vesicles. The ER appears to control both endosomal tubule formation and fission, involving different types of ER–endosome contact sites.

The formation of endosomal recycling tubules requires actin polymerization by the WASH complex, which is coupled to the cargo-sorting retromer machinery by its subunit FAM21 (Derivery et al., 2009; Gomez and Billadeau, 2009; Harbour et al., 2012; Puthenveedu et al., 2010). The transient accumulation of PtdIns4P on endosomes is coupled to a transient burst of WASH-dependent actin nucleation to facilitate retromer function, and the ER is the master regulator of these dynamics (Dong et al., 2016). A type II PI 4-kinase localizes to the WASH complex and produces a local pool of PtdIns4P (Ryder et al., 2013). Endosomal OSBP interacts with PtdIns4P via its PH domain and tethers the endosome to the ER by interacting with VAP-A/B (Fig. 1 3). Here, OSBP transfers PtdIns4P to the ER-resident lipid-phosphatase SAC1, which dephosphorylates PtdIns4P, ensuring the transient PtdIns4P pool on the endosome required for tubule dynamics. In addition, the PtdIns3P binding retromer subunit SNX2 interacts with the ER through VAP-A/B. As actin nucleation by WASH is tightly coupled to retromer-dependent cargo sorting, the ER presumably coordinates their activities through the interaction between VAP-A/B (ER), SNX2 (retromer), and OSBP/PtdIns4P (WASH), all of which localize to the same intracellular hotspots. When this mechanism is perturbed by the depletion of VAP-A/B, SNX2, or OSBP, both PtdIns4P and actin hyper-accumulate on endosomes, and the traffic between endosomes and the Golgi complex is disrupted (Dong et al., 2016). Thus, by regulating endosomal PtdIns4P levels, the ER affects WASH-dependent actin nucleation and retromer function; however, it remains to be seen how PtdIns4P mechanistically interacts with WASH activity.

In addition to regulating endosomal actin dynamics, the ER defines the position and timing of endosome fission (Hoyer et al., 2018; Rowland et al., 2014). Immediately prior to fission, contact sites are formed between ER tubules and endosome buds on sites marked by the WASH component FAM21. The organelles are tethered by the ER membrane protein TMCC1 and endosomal Coronin1C, which is connected to actin on the endosomal buds (Fig. 1 3). Both proteins are required for contact site formation and fission of WASH-containing endosome tubules. Depletion of TMCC1 disrupts recycling of the CI-MPR from endosomes to the Golgi to a similar extent as the depletion of FAM21 (WASH) or VPS35 (retromer), emphasizing the role of ER in this process (Hoyer et al., 2018). Coronin1C confines the localization of actin to bud necks, thereby defining membrane availability for ER–endosome contact sites (Striepen and Voeltz, 2022). How the ER promotes fission is, however, not understood. It will be important to investigate a possible connection with the PtdIns4P-regulated mechanism discussed above. It is tempting to speculate that the final fission step is facilitated by the ESCRT-III-related proteins, IST1 and CHMP1B, which are known to mediate positive membrane bending and constriction (Nguyen et al., 2020) and are connected to the ER by Spastin M1, which is indeed required for the fission of tubules and the recycling of endosomal cargo (Allison et al., 2013).

### Coordination of receptor dephosphorylation and formation of multivesicular endosomes

Upon growth factor stimulation, activated growth factor receptors, such as EGFR, are internalized by endocytosis for their final degradation in lysosomes, a process referred to as receptor downregulation (Huotari and Helenius, 2011; Scott et al., 2014). This process ensures that signaling is switched off in a timely manner to prevent hyperproliferation. To attenuate EGFR signaling, the receptors are dephosphorylated and sorted into forming intraluminal vesicles (ILVs) of multivesicular endosomes (MVEs) on their way to the lysosome. It is interesting to note that EGF-stimulation itself induces this process by triggering the dephosphorylation of EGFR and at the same time stimulates ILV formation. Intriguingly, the ER is recruited to promote both tasks.

The phospholipid-binding protein Annexin A1 associates with EGFR-containing MVEs, whereas its ligand S100A11 localizes to the ER (Futter et al., 1993; Gerke and Moss, 2002; Liu et al., 2012; Fig. 1, 4 a). EGFR-induced phosphorylation of Annexin A1 induces the formation of Annexin A1/S100A11-mediated ER–endosome contact sites (Eden et al., 2016). Both Annexin A1 and S100A11 are Ca^2+^ binding proteins, and the contact site formation is aided by the local increase in Ca^2+^, which is induced by the endosomal NAADP-sensitive two-pore Ca^2+^ channel TPC1 (Kilpatrick et al., 2017). These contact sites promote EGF-induced ILV formation (Eden et al., 2016; White et al., 2006; Wong et al., 2018). First, the protein tyrosine phosphatase 1B (PTP1B), which is embedded in the cytoplasmic face of the ER, dephosphorylates EGFRs on the endosomes, depending on Annexin A1/S100A11-mediated ER–endosome contact sites (Eden et al., 2010). At the same time, the EGFRs are sorted into forming ILVs by the ESCRT protein machinery, which interacts with the ubiquitinated EGFRs and mediates membrane deformation and scission to generate ILVs (Migliano et al., 2022). Interestingly, the ESCRT proteins HRS and STAM are dephosphorylated by PTP1B, implying that the Annexin A1/S100A11-mediated ER–endosome contact sites can regulate ESCRT function (Eden et al., 2010; Stuible et al., 2010). This functional relationship, which could facilitate the progression of cargo through downstream ESCRTs and ILV formation, needs further investigation.

In addition to acting on the ESCRT machinery through PTP1B, the Annexin A1/S100A11-mediated ER–endosome contact sites can facilitate ILV formation through a different mechanism. ILVs are rich in cholesterol, and high levels of endosomal cholesterol are required to form ILVs (Möbius et al., 2003). Cholesterol can be supplied by the uptake of low-density lipoprotein (LDL) by receptor-mediated endocytosis (Anderson et al., 1977). To fuel ILV formation in the absence of LDL, cholesterol needs to come from internal sources such as the ER. When endosomal cholesterol levels are low, EGF-stimulated ILV formation depends on Annexin A1/S100A11-mediated ER–endosome contacts (Eden et al., 2016). As Annexin A1/S100A11 does not harbor sterol transfer properties, such delivery has to be coordinated with a lipid transfer protein. The endosomal cholesterol sensor ORP1L localizes to Annexin A1-dependent ER–endosome contact sites in the absence of LDL and is a plausible candidate for this activity. When endosomal cholesterol levels are low, a conformational change exposes the ORP1L FFAT-motif, inducing binding to VAP-A in the ER (Rocha et al., 2009; Fig.1, 4 b). Here, ORP1L facilitates the transfer of cholesterol from the ER to EGFR-containing endosomes and stimulates ILV formation in a manner that requires its interaction with VAP-A (Eden et al., 2016). Whether ORP1L is directly responsible for transfer or regulates another lipid transfer protein is unresolved. It is interesting to note that under cholesterol depletion, the interaction of ORP1L with VAP-A in the ER at the same time leads to the loss of dynein and the HOPS complex from the endosomes (van der Kant et al., 2013). This will inhibit the perinuclear translocation and fusion of MVEs when cholesterol levels are low, and could thus halt the maturation of endosomes to ensure proper sorting of EGFRs into ILVs by use of cholesterol from the ER.

### Control of endosome maturation and homeostasis

To maintain lipid homeostasis, the ER influences the transport of lipids from the ER to endosomes and vice versa. In addition to the lipid transporters PDZD8 and ORP1L mentioned above, STARD3 resides in EGFR-negative endosomes and facilitates cholesterol transport from the ER to the endosomes, which are anchored to the ER through VAP-A/B and MOSPD2 (Alpy et al., 2013; Voilquin et al., 2019; Wilhelm et al., 2017). Conversely, NPC1 facilitates the transfer of cholesterol from endosomes to the ER by interaction with ORP5 in the ER (Fig.1, 4 c; Du et al., 2011; Raiborg et al., 2015b). The ER plays a pivotal role in maintaining endosome homeostasis and maturation by regulating endosomal identity (Wu and Voeltz, 2021), and the sorting and trafficking of hydrolytic enzymes and endocytosed proteins and lipids, as exemplified above. Mutations in NPC1 lead to the accumulation of cholesterol in endosomes, causing the neurodegenerative disease Nieman-Pick (Mukherjee and Maxfield, 2004). Accumulation of endocytosed or cellular material caused by dysfunctional ER–endosome MCS proteins can thus lead to severe metabolic and developmental defects, as manifested by genetic diseases, collectively termed lysosomal storage disorders (Platt et al., 2012).

## ER as source and regulator of autophagosomes

**Figure 2.**
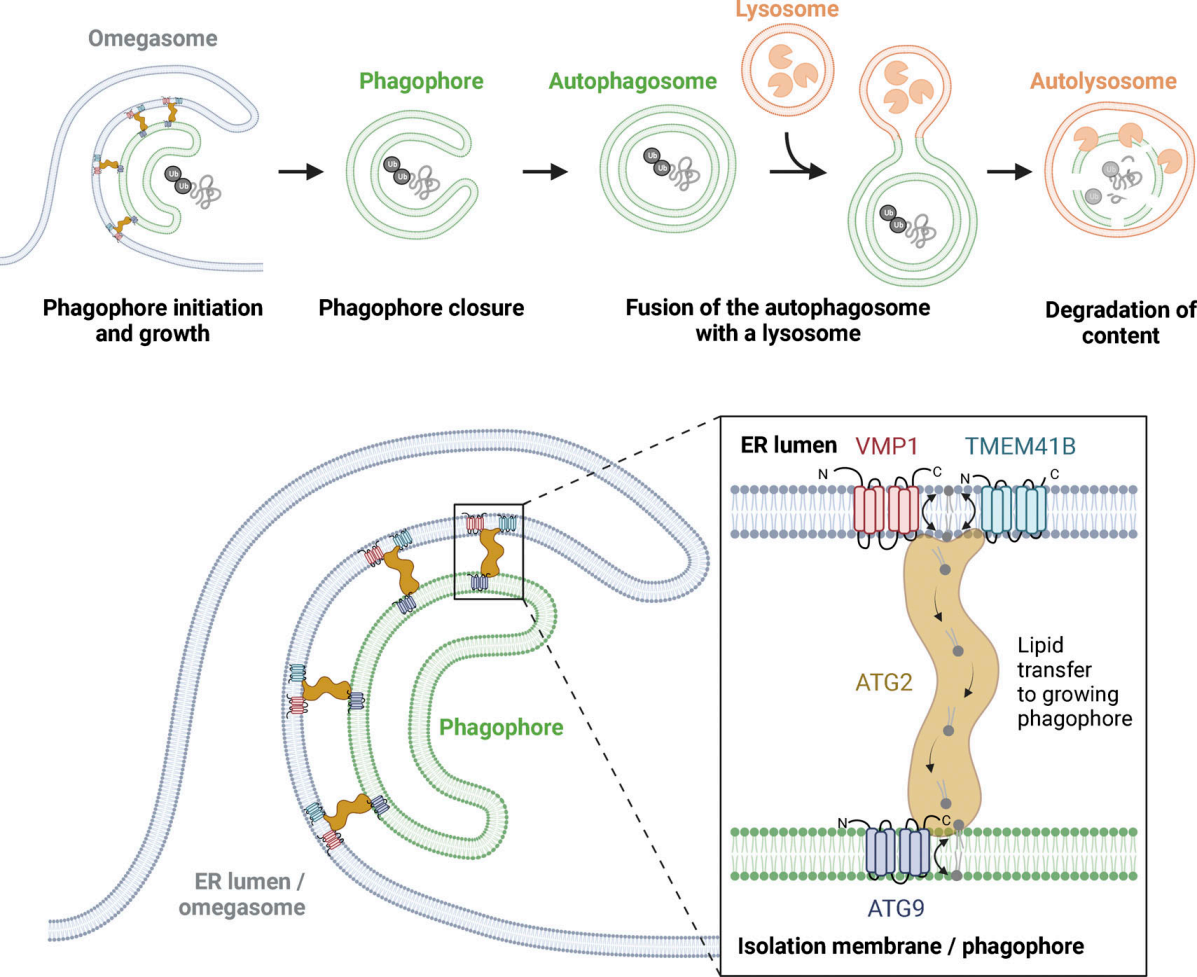
**Biogenesis of the phagophore membrane via ER contacts.** Autophagy is initiated by sequestration of cytoplasmic material by a double-membrane phagophore, whose seed is thought to be composed of ATG9-containing vesicles originating from the Golgi. The phagophore elongates and closes to form an autophagosome, and the sequestered material is degraded once the autophagosome fuses with a lysosome. Phagophore elongation is promoted by a flux of lipids from the ER to the phagophore membrane via the lipid channel transporter ATG2, which tethers subdomains of the ER to growing phagophores by interaction with the ER-localized lipid scramblases TMEM41B and VMP1, and the lipid scramblase ATG9 in the phagophore membrane (additional contacts between the membranes are likely). TMEM41B-VMP1 and ATG9 serve to maintain transbilayer lipid balance in the ER and phagophore membrane, respectively.

Macroautophagy (hereafter, autophagy) is a catabolic process that entails sequestration of portions of cytoplasm by a double-membrane structure known as the phagophore (Fig. 2). The phagophore closes to form an autophagosome, and when the autophagosome fuses with a lysosome to form an autolysosome, the sequestered material is degraded by lysosomal hydrolases (Melia et al., 2020; Mizushima and Komatsu, 2011) The catabolic functions of autophagy are used to supply cells with amino acids and other small molecules during conditions of low nutrient availability, but autophagy is also used to protect cells from potentially harmful cytoplasmic objects such as protein aggregates, pathogens, and damaged organelles.

### Biogenesis of the phagophore membrane

Although several cellular membranes have been proposed as the origin of phagophore membranes, there is little doubt that the ER is a major source (Lamb et al., 2013; Melia et al., 2020). The fact that autophagosome membranes, in contrast to other cellular membranes, are almost devoid of transmembrane proteins (Fengsrud et al., 2000) suggests that much of the phagophore could originate from de novo membrane synthesis rather than budding from existing membranes. In support of this, a large cytosolic protein required for autophagosome biogenesis, ATG2, is an elongated lipid transporter that contains a hydrophobic groove through which lipids can slide in an efficient way (Ghanbarpour et al., 2021; Maeda et al., 2019). ATG2 could thus function in MCSs that bridge the lipid-synthesizing ER and the forming phagophore.

ATG2 interacts with two lipid scramblases in the ER membrane, TMEM41B and VMP1, and with a lipid scramblase on Golgi-derived vesicles, ATG9 (Ghanbarpour et al., 2021; Judith et al., 2019; Noda, 2021). Lipid scramblases transfer lipids from one membrane leaflet to the other, and it has been proposed that even a single ATG9-containing vesicle might act as a seed for phagophore biogenesis (Ghanbarpour et al., 2021). In this model, ATG2 mediates lipid transport from the ER membrane to the seeding vesicle, whereas TMEM41B and VMP1 re-equilibrate the leaflets of the ER during lipid extraction. In the seed vesicle, ATG9 scrambles ER-derived lipids upon their delivery to allow phagophore expansion (Fig. 2). Even though this is an attractive model that explains the requirement for lipid transporters and scramblases in autophagosome biogenesis, it still needs to be verified experimentally. Hybrid organelles consisting of membranes from endosomes and the cis-Golgi have recently been put forward as precursors of phagophores (Kumar et al., 2021), and it remains plausible that autophagosomes can originate from membranes other than the ER, at least under some conditions (Melia et al., 2020).

Class III PI 3-kinase (PI3K-III), which phosphorylates PtdIns into PtdIns3P, is required for phagophore biogenesis, and it is conceivable that PtdIns3P contributes to defining the sites of phagophore initiation. Indeed, ATG14, a subunit of the autophagy-specific version of PI3K-III, localizes to ER sites, and this localization is required for autophagy (Matsunaga et al., 2010). The PtdIns3P-binding protein DFCP1 is recruited to PtdIns3P-containing ER subdomains in response to amino acid starvation, a classical way to induce autophagy, and is a likely PtdIns3P effector in autophagosome biogenesis. Due to their omega shape in light microscopy, DFCP1-containing ER subdomains are referred to as omegasomes (Axe et al., 2008). The exact spatial and functional relationships of omegasomes with phagophores are not known, but current evidence suggests that omegasomes could represent ER subdomains that are involved in the elongation and sculpting of the phagophore.

PtdIns3P is not only found on ER subdomains but also on phagophore membranes (Cheng et al., 2014), suggesting the involvement of additional PtdIns3P-binding proteins in phagophore biogenesis. PtdIns3P-binding proteins of the WIPI family are good candidates as they interact with ATG2 and localize to the growing phagophore. WIPI4, which shows the highest affinity to ATG2, binds to one of the tips of ATG2, consistent with the idea that ATG2 could be recruited by WIPI4 to form a lipid-transporting bridge between the ER and the tip of the phagophore (Chowdhury et al., 2018). However, the spatiotemporal relationships between ATG2, ATG9, PtdIns3P, DFCP1, and WIPI4 during phagophore biogenesis remain to be defined.

### Control of autophagosome fusion

Autophagy culminates in the fusion of autophagosomes with lysosomes. The membranes of late endosomes and lysosomes contain the small GTPase RAB7, and among the RAB7 effectors they recruit are ORP1L, RILP, and PLEKHM1. As described above, ORP1L is a cholesterol sensor that forms tripartite contacts with RAB7 and the dynein adaptor RILP, thereby promoting dynein-mediated transport of late endosomes and lysosomes toward the microtubule organizing center. The endolysosomal protein PLEKHM1, in concert with RILP, recruits the HOPS complex, which promotes fusion between lysosomes and autophagosomes (McEwan et al., 2015; Wijdeven et al., 2016). If the lysosomes have low cholesterol content, the FFAT motif of ORP1L is exposed and engages in interaction with VAP-A in the ER membrane. The cholesterol-free conformation of ORP1L not only prevents the interaction of RILP with dynein and HOPS, but also dissociates PLEKHM1, and this inhibits both lysosome motility toward the microtubule organizing center and fusion between lysosomes and autophagosomes (Wijdeven et al., 2016). Thus, autophagic flux is positively regulated by cholesterol and negatively controlled by the ER–lysosome MCSs. Since autolysosomes, like lysosomes, contain RAB7 and ORP1L, their motility is regulated in the same manner.

## Regulation of mitochondria by ER

MCSs between ER and mitochondria are guided by bridging proteins that tether the two membranes. Such MCSs are important for several mechanisms, including mitochondrial homeostasis, lipid composition, nutrient sensing, and regulation of the apoptotic machinery. The MCSs affect mitochondria both through physical interactions between the membranes, as with mitochondrial fission, and also via Ca^2+^ release and signaling, as for regulation of the Krebs cycle (Marchi et al., 2014; Rowland and Voeltz, 2012).

### Ca^2+^ transfer between ER and mitochondria

**Figure 3.**
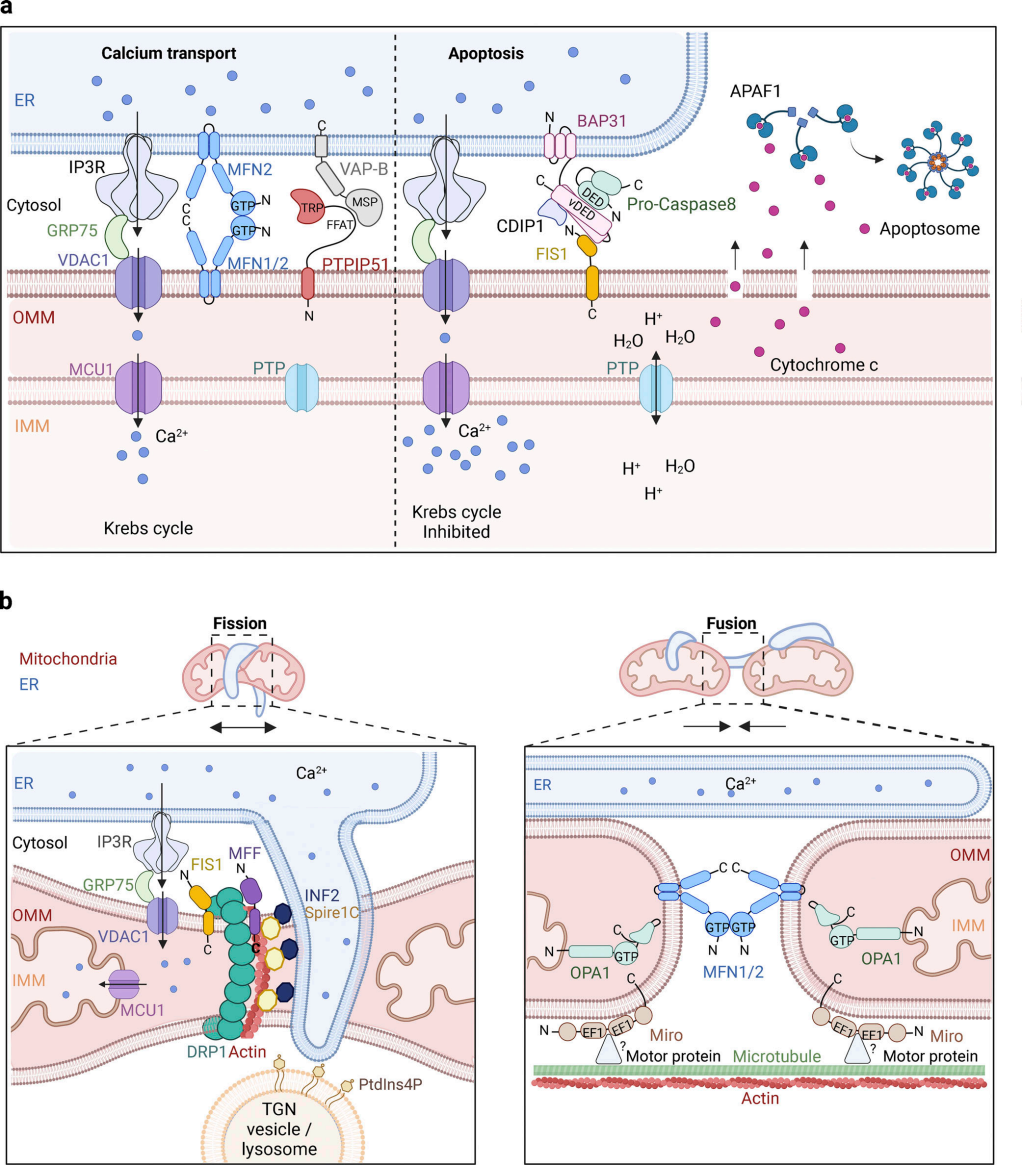
**Control of mitochondrial functions via contacts with ER.** The figure shows an overview of some of the best-studied functional contacts between the ER and mitochondrial membranes. **(a)** Calcium transport for homeostasis or apoptosis. In healthy cells, Ca^2+^ flows from the lumen of ER via the IP3R and through the VDAC1 channel in the outer mitochondria membrane (OMM). GRP75 binds both channels to stabilize the synapse. Inside the mitochondria, ions pass the inner mitochondria membrane (IMM) via MCU1 where Ca^2+^ is needed for the Krebs cycle. Several protein–protein interactions are required to strengthen the contact site. Examples of such contacts are the ER proteins MFN2 and VAP-B which can interact with mitochondria-resident proteins MFN1/2 and PTPIP51, respectively. During apoptosis, a membrane complex consisting of BAP31, procaspase-8, CDIP1, and FIS1 tethers mitochondria and ER together in addition to the complex required for calcium transport. BAP31 from the ER bind both CDIP1 and procaspase-8, the latter is activated by interacting via its DED domain to bind a vDED domain on BAP31. FIS1 on the mitochondria interacts with BAP31 to bridge the two organelles. These apoptotic cues lead to increased Ca^2+^ levels in the mitochondria matrix and open the PTP. This disrupts the proton gradient and eventually leads to swelling and rupture of the mitochondria membrane, allowing cytochrome c to leak into the cytosol. APAF1 binds cytochrome c and assembles the apoptosome to execute apoptosis. **(b)** Mitochondria fission and fusion. ER marks the position for mitochondria fission or fusion by wrapping tubules around the mitochondria. Spire1C nucleates actin filaments and binds INF2 on the ER. INF2 stimulates the mitochondrial Ca^2+^ uptake and polymerizes actin filaments to further connect ER and mitochondria, allowing the IMM to divide first. DRP1 self assembles into a spiral guided by MFF and FIS1, and with the help of actin filaments constricts to separate the OMM. The final separation of the mitochondria can be aided by lysosomes or trans-Golgi network vesicles containing PtdIns4P at the ER–mitochondria contact site. Fusion is engaged by homodimerization between MFN1 or MFN2 in the OMM through their GTPase domain. Similarly, the GTPase domain on OPA1 interacts to fuse the inner membranes. Miro can bind motor proteins on both microtubules and actin filaments, possibly to strengthen the ER–mitochondria contact by reducing mitochondria movements.

The ER contains a highly concentrated pool of intraluminal Ca^2+^, which is involved in the regulation of processes ranging from ATP production to the onset of apoptosis. Upon activation of the IP_3_ gated Ca^2+^ channel (IP3R), the ER can release Ca^2+^ ions to the surrounding milieu. The close proximity of the ER–mitochondria MCSs allows for a directional flow of Ca^2+^ to enter the mitochondria through the voltage-dependent anion channel 1 (VDAC1) in the outer membrane (Gincel et al., 2001; Rapizzi et al., 2002) and the mitochondrial calcium uniporter (MCU1) in the inner membrane (Kirichok et al., 2004). The glucose-regulated protein 75 (GRP75) bridges the two organelles to form a stable “synapse” for the Ca^2+^ transfer by binding both VDAC1 and IP3R (Szabadkai et al., 2006; Fig. 3 a). This synapse is necessary for mitochondrial function, homeostasis, energy production, and viability. The concentration of Ca^2+^ inside the inner mitochondrial membrane has consequences for ATP production through the regulation of Ca^2+^-dependent enzymes in the Krebs cycle (Rossi et al., 2019). However, excessive levels of Ca^2+^ ions can induce apoptosis (Rasola and Bernardi, 2011; see below).

Several protein–protein interactions are involved in the flow of Ca^2+^ between these membranes, likely due to its important functions in the regulation of both cell growth and cell death. One such example is VAP-B on ER, which binds PTPIP51 on the outer mitochondrial membrane to ensure proper Ca^2+^ release from ER lumen to the mitochondria (De Vos et al., 2012). The interaction is mediated via the FFAT-like motif on PTPIP51 and the MSP motif on VAP-B. A mutated version of VAP-B, VAPB^P56S^, and the dysregulation of Ca^2+^ flow between ER and mitochondria are both associated with amyotrophic lateral sclerosis, highlighting the physiological importance of this connection (Langou et al., 2010; Nishimura et al., 2004). Mitofusin (MFN) is another example of a protein bridge that supports Ca^2+^ transport from the ER to the mitochondria. The ER membrane carries MFN2, which can bind heterotypically or homotypically to MFN1 or MFN2, respectively, on the mitochondrial membrane. This interaction aids in forming a stable bridge between the organelles during mitochondria Ca^2+^ uptake (de Brito and Scorrano, 2008).

The ER-resident lipid transfer protein PDZD8 (described under “Synchronization of endosome translocation and lipid transfer”) is a mammalian paralog of the yeast protein Mmm1, a member of the ER–mitochondrial encounter structure (ERMES) complex, implicated in the formation of ER–mitochondria contact sites (Hirabayashi et al., 2017; Wideman et al., 2018). In neurons, PDZD8 is necessary for the contact between ER and mitochondria during Ca^2+^ transport. This MCS is proposed to be utilized by neurons to regulate dendritic excitability and plasticity during signal transduction. PDZD8 establishes directionality of Ca^2+^ flux from IP3R and ryanodine receptors toward the mitochondrion. In the absence of PDZD8, the cytosolic Ca^2+^ concentration increases. The protein partner of PDZD8 on the mitochondria membrane has yet to be elucidated (Hirabayashi et al., 2017).

### Regulation of apoptosis

In the context of cell death, the ER-resident protein BAP31 is crucial for ER–mitochondrial tethering (Ng et al., 1997), and several BAP31 complexes are associated with cell death, including FIS1 (Iwasawa et al., 2011) and CDIP1 (Namba et al., 2013; Fig. 3 a). These protein–protein contacts regulate cell death by establishing signaling platforms, translating apoptotic cues, and engaging in the onset of apoptosis (Iwasawa et al., 2011; Mattson and Chan, 2003; Namba et al., 2013). The initiation of cell death will activate either the intrinsic or extrinsic apoptotic pathway. However, a few common events occur independently of the mode of action. These include the activation of caspases by cleavage and the release of cytochrome c into the cytosol from the mitochondria. These actions are downstream of an increased flow of Ca^2+^ from ER into the mitochondria. When the concentration of Ca^2+^ reaches a certain threshold, the permeability transition pore (PTP) opens and allows for water molecules and protons to travel freely over the inner mitochondria membrane. This disrupts the proton gradient and induces swelling and rupture of the outer membrane (Halestrap, 2009). As a result, cytochrome c leaks into the cytosol where it binds the apoptotic protease-activating factor 1 (APAF1) machinery (Hardingham and Bading, 2003; Mattson and Chan, 2003), causing the apoptosome to assemble and execute apoptosis (Rasola and Bernardi, 2011).

Cell death can also be mediated through ceramide-induced apoptosis (Obeid et al., 1993). Ceramides synthesized by ER are highly regulated and are normally transported to the Golgi for further processing. A rise in ceramide levels followed by the recruitment of ceramide binding proteins on the outer mitochondria membrane can trigger apoptosis. The exact molecular mechanisms leading to this event remain elusive, but VDAC2 in the outer mitochondrial membrane has recently been shown to bind ceramides and acts as an effector of cell death signals (Dadsena et al., 2019).

Interestingly, BAP31 in the ER–mitochondria MCSs is not restricted to cell death signaling. The mitochondrial membrane protein TOM40 can bind BAP31 to recruit NDUSF4. This protein complex is involved in stress sensing and cellular homeostasis. The lack of BAP31 activates autophagy and glycolysis while reducing mitochondrial oxygen consumption (Namba, 2019). Thus, BAP31 has a role in ER MCS during both self-preservation and self-destruction.

### Mitochondrial fission

Homeostasis of mitochondria is maintained by fission and fusion of the organelle. ER–mitochondrial contacts spatially define where the mitochondrion will divide (Abrisch et al., 2020; Friedman et al., 2011), as ER tubules wrap around the mitochondria to indicate the position for fission (Abrisch et al., 2020; Chakrabarti et al., 2018; Korobova et al., 2013; Fig. 3 b). The ER-associated formin, inverted formin-2 (INF2), polymerizes actin filaments to establish a close contact between the two organelles. The INF2-mediated actin polymerization stimulates mitochondria Ca^2+^ uptake. Spire1c is an actin nucleator and resides on the outer mitochondrial membrane during fission. Here it binds INF2 directly to connect the two organelles, as well as initiating nucleation of the actin filaments (Manor et al., 2015). ER tubules in the MCS release Ca^2+^, which enters the mitochondria through the VDAC1 channel. INF2 has been implied in the constriction of the inner mitochondria membrane indirectly by an increase in ER–mitochondria contact sites following an influx of Ca^2+^, which triggers the inner mitochondrial membrane to divide first (Chakrabarti et al., 2018). It is unknown exactly how the inner membrane divides, but the electron transport chain is required for the execution. Constriction of the outer membrane depends on the cytosolic GTPase dynamin related-protein 1 (DRP1) to self-assemble into a ring around the mitochondrion at the fission site. For the Drp1-spiral to form, the outer membrane receptors FIS1 and MFF guide the assembly (Elgass et al., 2013; Koch et al., 2005). Drp1 binds actin filaments and modulates actin bundles in vitro (Ji et al., 2015). It is therefore possible that the actin filaments on the mitochondria surface are anchored directly to the DRP1 spiral. The spiral constricts with the aid of actin–myosin filaments (Smirnova et al., 2001). Thus, the inner membrane scission is followed by constriction of the outer membrane as the Drp1 spiral tightens and ultimately results in the formation of two daughter organelles (Chakrabarti et al., 2018; Korobova et al., 2013).

A recent contribution to the field of mitochondrial fission is the observation that PtdIns4P-containing vesicles derived from lysosomes or the TGN interact with ER–mitochondria MCSs, forming three-way contacts (Boutry and Kim, 2021; Nagashima et al., 2020). Within these contact sites, ORP1L was suggested to transfer PtdIns4P from lysosomes to the mitochondrion, promoting mitochondrial fission (Boutry and Kim, 2021). Likewise, inhibition of the formation of PtdIns4P microdomains on TGN vesicles results in branched and hyperfused mitochondria (Nagashima et al., 2020), thus indicating an important role of ER–mitochondria triple contact sites to finalize fission.

When mitochondria divide, the daughter mitochondria must bear a copy of the mitochondrial DNA (mtDNA) found in the parental mitochondrion. Subpopulations of ER–mitochondria contact sites have been shown to be specifically reserved for and required for the synthesis of mtDNA (Lewis et al., 2016). Following duplication of the nucleoid, DRP1 regulates the mtDNA synthesis and the distribution to the daughter organelles during fission by altering the ER sheets that contact the mitochondria (Ilamathi et al., 2022; Lewis et al., 2016).

### Mitochondrial fusion

It has been proposed that mitochondrial fission and fusion events are coordinated to quickly respond to metabolic cues. Hence, as for mitochondrial fission, the ER marks the sites of mitochondrial fusion (Guo et al., 2018), and molecules involved in fusion and fission colocalize in ER–mitochondria contact sites (Abrisch et al., 2020). It has been proposed that ER tethering guides the position and timing of mitochondria fusion, but the exact role of ER–mitochondria MCS is still not clear (Gao and Hu, 2021). Fusion of the outer mitochondria membranes is executed by the mitofusins, MFN1 and MFN2 (Cao et al., 2017; Chen et al., 2003), while Opa1 regulates the fusion of the inner mitochondria membrane (Song et al., 2009; Fig. 3 b). Even though both hetero- and homotypic protein interactions can occur between MFN1 and MFN2, it is the homotypic interactions that are required for fusion (Chen et al., 2003). Interestingly, only a certain protein conformation of mitofusins allows for mitochondrial fusion (Franco et al., 2016). Both MFN1 and DRP1 puncta localize to the ER–mitochondria contact sites during the synchronized fusion and fission events, respectively (Abrisch et al., 2020). To maintain the ER–mitochondria contact during fusion, the Ca^2+^ sensitive molecule Miro will decrease mitochondria motility (Kornmann et al., 2011). Miro is also a motorprotein-adaptor involved in both actin filament and microtubule transport. It is unknown exactly how Miro regulates the translocation of mitochondria during fission and fusion; however, acetylation of Miro at specific Lysine residues has been implicated in the context of mitochondria transportation in neurons (Kalinski et al., 2019). More research is needed to further explore the role of Miro in mitochondria fission and fusion.

### Lipid transfer between ER and mitochondria

Mitochondria rely on lipid transport proteins to maintain membrane homeostasis. The evolutionarily conserved ER protein LTC1 is found at MCSs between mitochondria and ER, depending on the mitochondrial import receptors Tom70/71. It has been suggested that the function of LTC1 is to transport and/or sense sterols to maintain correct lipid homeostasis and organelle function (Murley et al., 2015).

Miro is not only involved in mitochondria mobility as discussed above but it also promotes lipid transfer. In this role, Miro has been shown to locate to the outer mitochondrial membrane to recruit the lipid channel transporter VPS13D, which is anchored to the ER by VAP-B. Analogously, Miro regulates lipid transfer in ER–peroxisome contact sites (Guillen-Samander et al., 2021; Kornmann et al., 2011).

The interaction between the ER-resident protein VAP-B and the mitochondrial protein PTPIP51 is also important for lipid transfer (De Vos et al., 2012). The interaction between the proteins is provided by a FFAT-like motif on PTPIP51 and an MSP domain on VAP-B. The contact site can provide the transport of phosphatidic acid from ER to mitochondria via a TRP motif in PTPIP51 (Yeo et al., 2021). Interestingly, the same protein–protein interaction and MCS are also involved in regulating autophagy in mammalian cells. Loss of PTPIP51 or VAP-B results in increased autophagy, while the overexpression of either protein reduces the number of autophagic structures, likely in a Ca^2+^-dependent manner (Gomez-Suaga et al., 2017).

## Regulation of peroxisomes by ER

**Figure 4.**
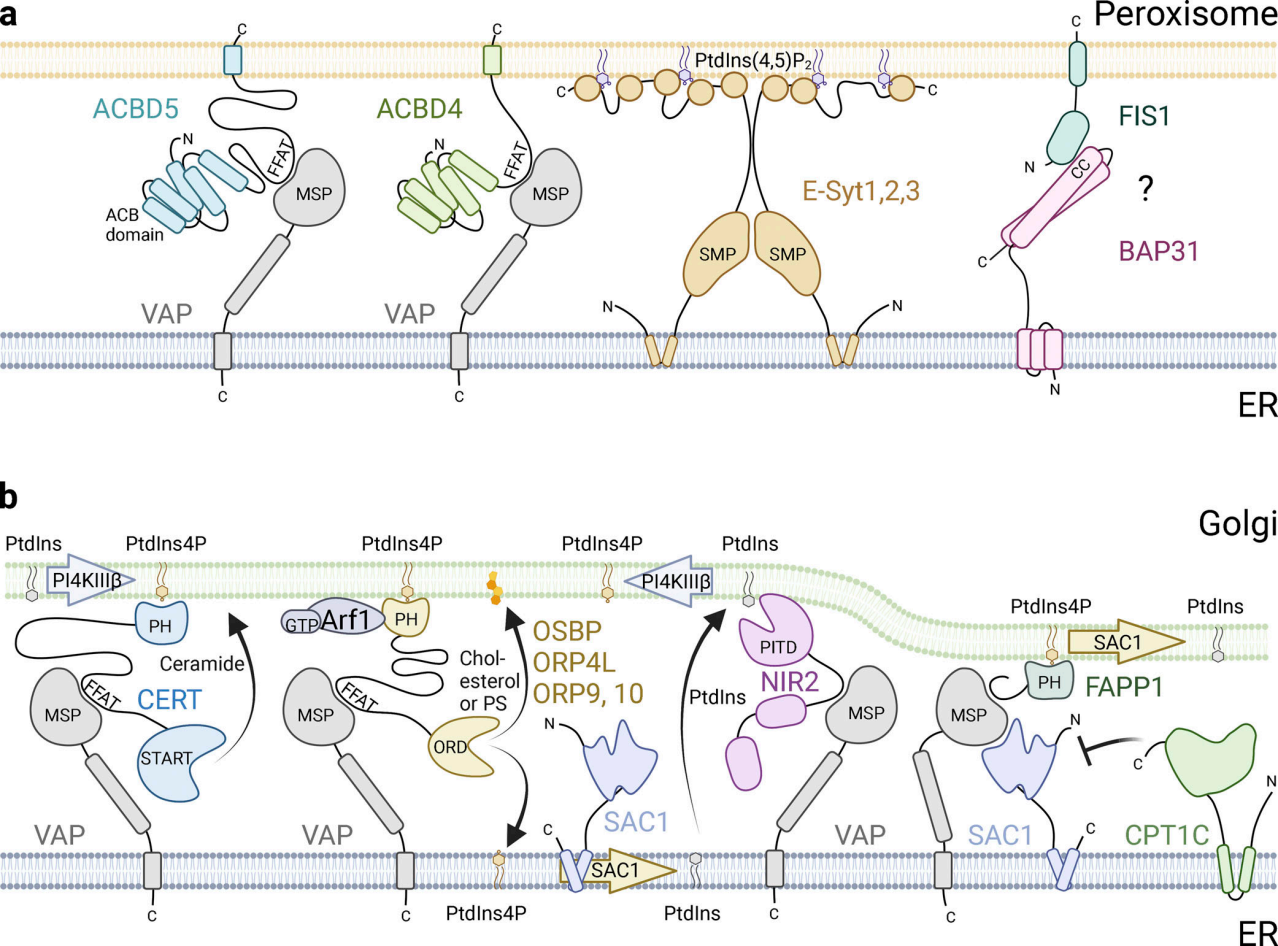
**Contact sites with ER control peroxisome and Golgi functions. (a)** The peroxisomal proteins ACBD4 and ACBD5 interact with the VAP proteins in the ER via their FFAT motifs, anchoring peroxisomes to the ER to facilitate lipid transfer. ER anchored E-Syts contact peroxisomal PtdIns(4,5)P_2_ to allow cholesterol transport from lysosomes via peroxisomes to the ER. The ER protein BAP31 can potentially interact with FIS1 on peroxisomes, presumably required for peroxisome fission similar to mitochondria. **(b)** PtdIns4P, the signature phosphoinositide of the Golgi. A PtdIns4P gradient is maintained by phosphorylation of PtdIns by PI4KIIIβ in the TGN and dephosphorylation of PtdIns4P in the ER by the phosphatase SAC1. CERT recognizes PtdIns4P in the Golgi and is tethered to the ER by binding to VAP, where it uses its START domain to transfer ceramide from the ER to the trans-Golgi network. OSBP and OSBP-related proteins (ORPs) interact with PtdIns4P in the Golgi and VAP in the ER. Here, the transfer of PtdIns4P from the Golgi to the ER along the PtdIns4P gradient fuels the counter-transfer of cholesterol or PS to the Golgi. NIR2 binds and transfers PtdIns from the ER to the Golgi, thereby replenishing the PtdIns pool. FAPP1 promotes the activity of SAC1 to dephosphorylate PtdIns4P in trans in narrow membrane contact sites. CPT1C inhibits SAC1 activity to maintain normal levels of PtdIns4P in the Golgi under basal conditions.

Peroxisomes are organelles with diverse metabolic tasks, such as fatty acid turnover, lipid synthesis, and the generation of reactive oxygen species (He et al., 2021; Mast et al., 2020). These processes require a close interplay with other cellular organelles, in particular with the ER. ER and peroxisome interactions have long been observed in electron micrographs, where peroxisomes are localized in the vicinity or even enwrapped by ER (Fahimi et al., 1993; Grabenbauer et al., 2000; Novikoff and Novikoff, 1972; Zaar et al., 1987). The molecular composition and function of peroxisome–ER tethers involve the peroxisomal C-tail anchored proteins acyl-CoA binding domain proteins 4 and 5 (ACBD4 and ACBD5), which interact with the VAP-A and VAP-B proteins in the ER via their FFAT motifs (Costello et al., 2017a; Costello et al., 2017b; Hua et al., 2017; Fig. 4 a). The interaction between ACBD5 and VAP-A/B regulates the extent of ER–peroxisome contacts (Costello et al., 2017a; Hua et al., 2017). Disruption of the tether by depletion of either ACBD5 or VAP-A and VAP-B increased peroxisome motility, indicating that the MCS acts like an anchor for the peroxisomes to the ER (Costello et al., 2017a; Wang et al., 2018). Further, elongation and growth of the PO membrane are reduced, which implicates this contact in lipid transfer. Indeed, plasmalogen and cholesterol homeostasis was shown to be disrupted when the MCS was compromised (Hua et al., 2017). Not surprisingly, a disruption of the peroxisome–ER contact site is associated with pathologies in mice and humans: loss or mutation of ACBD5 causes retinal dystrophy and white matter disease, which are characterized by an increase in very long-chain fatty acids due to impaired lipid transfer and impaired peroxisomal β-oxidation (Bartlett et al., 2021; Darwisch et al., 2020; Ferdinandusse et al., 2017; Gorukmez et al., 2022; Hua et al., 2017; Yagita et al., 2017). Interestingly, more levels of regulation of MCS formation are currently identified, such as phosphorylation of FFAT motifs, which can either promote or inhibit contact formation: ACBD5 phosphorylation through GSK3b was shown to negatively regulate the ACBD5–VAP–B interaction and thus peroxisome–ER MCS formation, while phosphorylation of the STARD3 FFAT motif induces contact formation with the MSP domains of VAP–A and –B (Di Mattia et al., 2020; Kors et al., 2022).

Another peroxisome–ER contact site is formed by ER-resident extended synaptotagmins (E-Syts-1, 2, and 3) which contact peroxisomal phosphatidylinositol-4,5-bisphosphate to allow cholesterol transport from lysosomes via peroxisomes to the ER (Xiao et al., 2019; Fig. 4 a).

Likely, more mammalian ER–peroxisome contact sites will be discovered in the future. MOSPD2 has been suggested to function as an alternative to VAP–A/B, since it also contains an MSP domain shown to interact with FFAT motif proteins (Di Mattia et al., 2018); however its interaction with ACBD4 or 5 has not yet been experimentally proven (Schrader et al., 2020). Further, the ER protein BAP31 interacts with the mitochondrial protein FIS1, which is required for mitochondrial fission (Fig. 4 a). As FIS1 and other mitochondrial fission proteins (DRP1 and MFF) can also be found on peroxisomes, and ER–mitochondrion or ER–endosome MCSs have been shown to mark fission sites (Friedman et al., 2011; Rowland et al., 2014), an analogous ER–peroxisome contact site might assist in peroxisome fission.

## ER-mediated regulation of the Golgi

Due to their collaborative roles in synthesis, modification, and transport of biomolecules, the ER and the Golgi require efficient ways to exchange molecules. Besides vesicular transport between the ER and cis-Golgi, biomolecules can also be exchanged directly at contact sites. To form contacts, PtdIns4P, the signature phosphoinositide of the Golgi, is indispensable as it governs the localization and regulation of lipid-exchange molecules.

### PtdIns4P fuels lipid transfer between ER and Golgi

PtdIns4P is generated by the Golgi-localized phosphatidylinositol kinase 4β (PI4KIIIβ) through the phosphorylation of PtdIns (Balla and Balla, 2006). Oxysterol-binding protein (OSBP) and ORPs (see text box), such as ORP4L, ORP9, and ORP10, are lipid-exchange transporters, which depend on high levels of PtdIns4P in the Golgi, both to form ER–Golgi contact sites and to function in lipid transport. These cytosolic transport proteins contact the Golgi with their PH domain, which binds PtdIns4P and ARF1-GTP, and they tether to the ER via their VAP-binding FFAT motifs, which allows the exchange of PtdIns4P against sterols or PS (Mesmin et al., 2013; Ngo and Ridgway, 2009). The lipid transfer is mediated by the ORD domain and requires PtdIns4P in exchange. The high PtdIns4P levels in the Golgi fuel a counter-transfer of cholesterol (OSBP, ORP4L, and ORP9) or PS (ORP10) to the Golgi (Maeda et al., 2013; Pietrangelo and Ridgway, 2018; Venditti et al., 2019b; Fig. 4 b). The PtdIns4P gradient is maintained by PI4KIIIβ-mediated synthesis of PtdIns4P in the TGN and dephosphorylation of PtdIns4P in the ER by the phosphatase SAC1 (Mesmin et al., 2013; Mesmin et al., 2017).

### SAC1 regulates PtdIns4P levels

SAC1 is an ER-resident phosphatase that dephosphorylates PtdIns4P in cis in the ER (Mesmin et al., 2013). However, close contact at ER–Golgi–MCS may allow SAC1 to also act in trans to consume PtdIns4P in the Golgi (Manford et al., 2010), and this activity is promoted by the presence of phosphatidyl-four-phosphate-adaptor-protein-1 (FAPP1) within these contacts (Venditti et al., 2019a; Fig. 4 b). Another regulator of SAC1 phosphatase activity is the neuronally-expressed CPT1C (Sierra et al., 2008), which cooperates with Protrudin as described above. CPT1C senses metabolic changes through binding to malonyl-CoA, an intermediate in de novo long-chain fatty acid synthesis, whose levels correlate with the nutritional state. Under basal conditions, CPT1C inhibits SAC1 activity to maintain normal levels of PtdIns4P in the Golgi, allowing AMPA receptor trafficking to the plasma membrane. Under glucose deprivation, CPT1C releases SAC1 inhibition allowing SAC1 to dephosphorylate PtdIns4P at ER–Golgi–MCS in trans, which results in AMPA receptor retention at the TGN (Casas et al., 2020). In this way, the ER can affect neuronal function and cognition depending on energy status.

### Sphingolipid transfer between ER and Golgi

PtdIns4P also plays an important role for sphingolipid transporters: Ceramide transfer protein (CERT) uses its START domain to transfer ceramide from the ER to the TGN for further processing into sphingomyelin (Hanada et al., 2003; Fig. 4 b). CERT forms a contact site by binding to ER-resident VAP proteins via its FFAT motif and its PH domain recognizes PtdIns4P in the Golgi (Hanada et al., 2003; Peretti et al., 2008). The activity of CERT is regulated by phosphorylation and by a negative feedback loop recognizing elevated DAG levels, resulting from sphingomyelin synthesis (Fugmann et al., 2007; Kumagai et al., 2014; Saito et al., 2008). In addition, the START domain can compete with the PH domain for PtdIns4P binding. When the levels of ceramide are high, the START domain is occupied by ceramide, and the shuttling of ceramide from ER to Golgi will occur. When the levels of ceramide are low, the START domain will bind to the PH domain, removing CERT from the Golgi (Prashek et al., 2017).

Phosphatidylinositol-four-phosphate adapter protein 2 (FAPP2; also known as PLEKHA8) possesses a PtdIns4P- and ARF1-GTP-binding PH domain at the N-terminus and a glycolipid transfer protein homology domain at the C-terminus, responsible for glucosylceramide (GlcCer) transport (Godi et al., 2004). Depletion of FAPP2 disrupts GlcCer transport from cis- to trans-Golgi, resulting in a disturbed synthesis of complex glycosphingolipids (D'Angelo et al., 2007; D'Angelo et al., 2013; Halter et al., 2007). How FAPP2 aids in the transport of GlcCer from the cis to the trans-Golgi is not yet fully understood, but may involve ER–Golgi contact sites: FAPP2 has a putative FFAT motif (Backman et al., 2018) and has been suggested to transfer GlcCer retrogradely to the ER, where GlcCer translocates into the lumen. From the ER lumen, GlcCer could be anterogradely transported to the trans-Golgi for further glycosylation into complex glycosphingolipids (Halter et al., 2007). Alternatively or additionally, FAPP2 may mediate the direct transfer of GlcCer from cis- to trans-Golgi (D'Angelo et al., 2007).

### Control of Golgi homeostasis

The phosphatidylinositol-transfer protein NIR2 (PYK2 N-terminal domain- interacting receptor 2) contacts the ER through a classical FFAT motif and it has a PtdIns-transfer domain (PITD) that mediates Golgi localization (Amarilio et al., 2005; Kim et al., 2013). Through its PITD domain, NIR2 transfers PtdIns from the ER to the Golgi, and thereby replenishes the substrate for the PtdIns4-kinase. This closes the PtdIns4P cycle of phosphorylation, transfer, and dephosphorylation, which is necessary to fuel sterol and PS transport (Fig. 4 b). In addition, NIR2 has been shown to regulate DAG levels at the Golgi apparatus (Litvak et al., 2005) and it can affect OSBP and CERT localization and activity (Peretti et al., 2008). The action of the lipid-transfer proteins OSBP, CERT, and NIR2 is thus intricately connected and coordinated at ER–Golgi contact sites.

Maintaining lipid homeostasis through lipid-transfer proteins is important for the physiologic function of the secretory pathway. Disturbances in lipid exchange and the resulting imbalances in PtdIns4P or cargo lipids disrupt Golgi morphology, lipid modifications, and anterograde cargo transport processes (Cruz-Garcia et al., 2013; Godi et al., 2004; Litvak et al., 2005; Peretti et al., 2008; Szentpetery et al., 2010; Wakana et al., 2021; Wakana et al., 2015). The ER maintains this balance not only through its direct function at lipid-transfer contact sites but also by anchoring TGN vesicles in the perinuclear area through T6BP/TAX1BP1–SQSTM1/p62–RNF26 as described above (Fig. 1, 1).

## Conclusions and perspectives

The ER controls the synthesis and trafficking of molecules not only through its classical functions in protein biosynthesis but also directly through membrane contacts with other organelles. The wide distribution of the ER throughout the cytoplasm makes it well suited to control other organelles via MCSs. As exemplified in this review, ER MCSs have diverse functions that include lipid transfer, Ca^2+^ transfer, protein and lipid dephosphorylation in trans, energy sensing, and regulation of organelle fusion, fission, motility, and positioning. This means that MCSs should always be taken into account when investigating organelle biology. Likewise, organelle-associated diseases can sometimes be understood by considering the dysfunctions of specific ER MCSs. Disruption of ER-organelle MCSs can severely affect cellular homeostasis, causing diseases ranging from metabolic and developmental defects, lipid storage, and neuronal diseases to cancer (Castro et al., 2018; Henne, 2017; Schrader et al., 2020; Simoes et al., 2020; Xu et al., 2020).

Even though we are getting a clearer picture of the protein compositions of many MCSs, most of them have not been characterized in full, and we know little about how the many different MCSs influence each other. Triple contacts of ER with endolysosomes, mitochondria, peroxisomes, Golgi apparatus, and lipid droplets have been described (Boutry and Kim, 2021; Elbaz-Alon et al., 2020; Guillen-Samander et al., 2021; Joshi et al., 2018; Nagashima et al., 2020), and it is conceivable that more tripartite MCSs will be detected as more are being characterized. The recent discovery of lipid channel proteins such as VPS13D and ATG2, which allow efficient lipid transport from the ER to other organelles via MCSs, has highlighted the involvement of MCSs in the composition and expansion of organelle membranes. Further progress in the burgeoning research field of ER MCSs will be spurred by combinations of molecular biological dissections of MCSs, structural analyses by cryo-electron microscopy, intracellular localization by advanced light and electron microscopy, and functional characterization by genetic approaches.
